# High-Mobility Group Box 1 Contributes to Cerebral Cortex Injury in a Neonatal Hypoxic-Ischemic Rat Model by Regulating the Phenotypic Polarization of Microglia

**DOI:** 10.3389/fncel.2019.00506

**Published:** 2019-12-11

**Authors:** Yanyan Sun, Mingyan Hei, Zhihui Fang, Zhen Tang, Bo Wang, Na Hu

**Affiliations:** ^1^Department of Pediatrics, The Third Xiangya Hospital of Central South University, Changsha, China; ^2^Neonatal Center, Beijing Children’s Hospital, Capital Medical University, Beijing, China; ^3^Department of Nuclear Medicine, The Second Xiangya Hospital of Central South University, Changsha, China

**Keywords:** hypoxic-ischemic (HI), HMGB1, microglia, polarization, cerebral cortex injury, neonatal

## Abstract

Neonatal hypoxic-ischemic (HI) encephalopathy is a severe disease for which there is currently no curative treatment. Recent evidence suggests that high-mobility group box 1 (HMGB1) protein can promote neuroinflammation after stroke in adult rodents, but its role in perinatal hypoxic-ischemic brain damage (HIBD) remains largely uninvestigated. In the present work, the potential role of HMGB1 in the pathogenesis of HIBD was explored. A HIBD model was established in postpartum day 7 rat pups. HMGB1 expression, the cellular distribution of HMGB1, and microglial activation were all evaluated. Glycyrrhizin (GL), an inhibitor of HMGB1, was used to investigate whether the inhibition of HMGB1 modulated microglial M1/M2 polarization or attenuated brain damage after HI. HAPI microglial cells and primary neurons were cultured *in vitro* and an oxygen-glucose deprivation model was established to evaluate the effects of different microglial-conditioned media on neurons using GL and recombinant HMGB1. Results showed that the expression of HMGB1 was increased in both the ipsilateral cortex and peripheral blood 72 h after HI. Immunofluorescence analyses showed that HMGB1 in the cortex was primarily expressed in neurons. This increase in cortical HMGB1 expression 72 h after HI was characterized by increased co-expression with microglia, rather than neurons or astrocytes. The expression of both M1 and M2 microglia was upregulated 72 h after HI. The administration of GL significantly suppressed M1 microglial polarization and promoted M2 microglial polarization. Meanwhile, GL pretreatment significantly alleviated brain edema and cerebral infarction. *In vitro* experimentation showed that HMGB1-induced M1-conditioned media aggravated neuronal damage, but this effect was neutralized by GL. These findings suggest that HMGB1 may result in an imbalance of M1/M2 microglial polarization in the cortex and thus cause neuronal injury. Pharmacological blockade of HMGB1 signaling may attenuate this imbalanced polarization of microglia and thus could be used as a therapeutic strategy against brain injury in HIBD.

## Introduction

Although neonatal resuscitation has been strongly promoted in recent years, moderate to severe hypoxic-ischemic brain damage (HIBD) still occurs after asphyxia (Barkhuizen et al., 2017). The pathogenesis of HIBD is complex, and there currently exists no effective treatment except for hypothermia. Even with timely hypothermia, more than 40% of neonates who experience severe asphyxia in the perinatal period still exhibit adverse outcomes, or even die as a result (Wu et al., 2016).

The expression of pro-inflammatory factors is higher in the developing brain than in the mature brain (Kaur et al., 2017). Given that HIBD is a pathological condition of the developing brain, it is hypothesized that inflammation, primarily mediated by microglia, may play an important role in HIBD after perinatal asphyxia. Microglia-related neuroinflammation is reported to be associated with the release of cytokines and additional inflammatory mediators, leading to secondary neuronal injury (Umekawa et al., 2015). Microglia can be classified into two main forms: M1-type (pro-inflammatory) and M2-type (anti-inflammatory) (Colton, 2009). M1 microglia promote neuronal death by expressing pro-inflammatory mediators, such as interleukin-6 (IL-6), tumor necrosis factor-α (TNF-α), and inducible nitric oxide synthase (iNOS). In contrast, M2 microglia promote tissue repair and support neuronal survival through the secretion of anti-inflammatory cytokines such as transforming growth factor-β (TGF-β), interleukin-10 (IL-10), and arginase-1 (Olah et al., 2011; Patel et al., 2013). Normally, there exists a careful balance of pro-inflammatory M1 and anti-inflammatory M2 microglia (Jin et al., 2019). Both *in vitro* and *in vivo* experiments have proved that correction of a polarization imbalance of M1/M2 microglia can inhibit the release of pro-inflammatory cytokines and decrease neurotoxicity (Weinstein et al., 2010; Xia et al., 2015; Zhou et al., 2019). *In vivo* studies have reported an imbalance of microglial M1/M2 polarization after hypoxic-ischemic (HI) exposure (Weinstein et al., 2010; Bhalala et al., 2014). However, the exact mechanism underlying this M1/M2 microglia imbalance after HI in neonatal models remains unclear.

High-mobility group box 1 (HMGB1) is a chromatin-associated protein widely expressed in the nuclei of brain cells, which, under physiological conditions, promotes neurite outgrowth and thus brain development (Merenmies et al., 1991; Guazzi et al., 2003; Liu et al., 2010). Under pathological conditions, HMGB1 can act as a pro-inflammatory factor, promoting brain damage (Wang et al., 1999; Zhang et al., 2011; Andersson et al., 2018). Studies have demonstrated that HMGB1 is involved in the pathogenesis of ischemic stroke in adult rodents, activating microglia and promoting neuroinflammation (Ye et al., 2019). In immature animal models, it was reported that HMGB1 translocated from nuclear to cytosolic compartments after HI (Zhang et al., 2016), and the translocation of HMGB1 was primarily in neurons along with release from apoptotic cells (Chen et al., 2019). This translocation may enable the action of HMGB1 as a proinflammatory cytokine that contributes to HI injury in the developing brain (Zhang et al., 2016). Unfortunately, the above studies mainly explored the cellular localization changes of HMGB1 after HI, further mechanism by which HMGB1 aggravates brain injury in HIBD is still unclear.

The objective of the present study was to explore whether HMGB1 played an important role in regulating the phenotypic balance of M1/M2 microglia in the cortex of neonatal SD rats after HI exposure, and whether the HMGB1 inhibitor, glycyrrhizin (GL), could modulate microglial M1/M2 polarization after HI *in vivo* and *in vitro*. We found that HMGB1 was upregulated in microglia after HI. Furthermore, HMGB1 was able to regulate the M1/M2 phenotypic polarization of microglia, leading to cortical injury.

## Materials and Methods

### Animals and Ethical Permission

All experiments were performed in accordance with the guidelines for experimental animal use of Central South University. The protocol was approved by the ethics committee of the Third Xiangya Hospital of Central South University (No. 2016-S006). Perinatal Sprague-Dawley (SD) rats were purchased from Central South University (China). All rats were housed in a 12-h light/dark cycled facility with free access to food and water.

### HIBD Animal Model and Drug Administration

The Rice and Vannucci HIBD model was induced with minor modifications in postpartum day 7 (P7) rat pups of both genders. In brief, rat pups were anesthetized with isoflurane and the left common carotid artery was permanently ligated with 5–0 silk sutures. Pups were returned to the dam for a 2 h recovery before initiation of a 2 h hypoxia exposure (37°C, 8% O_2_/92% N_2_). The sham-operation control animals were given only a small incision on the left side of the neck and the left common carotid artery was isolated without artery ligation or hypoxia treatment.

SD rat pups were randomly divided into three groups: sham + PBS, HI + PBS, and HI + GL. In the HI + GL group, GL was administered 1 h before artery ligation (20 mg/kg, i.p.). In the other two groups, an equal volume of 0.01M PBS was administered.

### Primary Neuron Cultures and Microglial-Conditioned Media Treatment

Cells from the microglia-like cell line HAPI (highly aggressive proliferating cell type) were seeded into 6-well plates at 1 × 10^5^/mL and incubated overnight in high-glucose Dulbecco’s Modified Eagle Medium (DMEM) containing 10% fetal bovine serum (FBS). Then, an oxygen-glucose deprivation (OGD) model was established to mimic the *in vivo* HI process. HAPI cells were divided into the following three groups: OGD + PBS, OGD + GL, and OGD + r-HMGB1. In brief, PBS (0.01M), GL (55 μM), and recombinant HMGB1 (r-HMGB1, 10 ng/mL) were added to the respective cell groups for 2 h. The cells’ medium was then replaced with glucose-free Earle’s balanced salt solution, and they were placed in an oxygen- deprived incubator (93% N_2_/5% CO_2_/2% O_2_) at 37°C for 12 h. Finally, the culture supernatant was collected, one part was used for an ELISA, and the other was used as a conditioned medium (CM) for primary neurons.

Primary cortical neurons were obtained from P1 rat pups. In brief, the cortices of P1 rats were isolated, digested by trypsin, and filtered using a 50 μm sterile nylon filter. Cells were then placed in 24-well plates pre-coated with poly-L-lysine in a neurobasal medium with 10% FBS and B27 supplement. The cells were placed in an incubator (37°C, 5% CO_2_) to differentiate for 7 days. At this point, the neuronal medium was removed and substituted with the aforementioned CM from HAPI microglial cells. To analyze the effects of CM on cell viability, neurons were cultured with this microglial CM for 24 h.

### Cell Viability and Neurite Length Measurement

Primary cortical neurons were seeded in 96-well plates at a density of 5 × 10^3^ cells/well. After culturing with microglial CM for 24 h, a cell viability assay was performed using a cell-counting kit (CCK-8; Beyotime, China). The optical density was read at a wave length of 450nm using an EnVision Xcite microplate reader (PerkinElmer, United States). Cell viability was calculated using the following formula:

$$Cellviability\left(\%\right)=\frac{o\,p\,t\,i\,c\,a\,l\,d\,e\,n\,s\,i\,t\,y\,o\,f\,t\,r\,e\,a\,t\,e\,d\,g\,r\,o\,u\,p}{o\,p\,t\,i\,c\,a\,l\,d\,e\,n\,s\,i\,t\,y\,o\,f\,c\,o\,n\,t\,r\,o\,l\,g\,r\,o\,u\,p}\times 100$$

For neurite length measurement, three digital images were taken per well after microtubule-associated protein 2 (MAP-2) and 4′, 6′- diamidino-2-phenylindole (DAPI) immunostaining. Using the plugins NeuronJ and ImageScience, Image J software was then used to calculate the average neurite length of MAP-2- positive neurons in every image following the instructions provided.

### Western Blotting

Western blotting was used to assess the expression of HMGB1, ionized calcium-binding adaptor molecule 1 (Iba-1), and β-actin in the cortex. Briefly, frozen cortex samples were completely homogenized in lysis buffer containing Phenylmethanesulfonyl fluoride (PMSF, Beyotime, China, ST505) and Radio Immunoprecipitation Assay (RIPA, Beyotime, China, P0013B) and centrifuged at 12,000 rpm for 15 min at 4°C. The supernatant was collected and contained the total protein extracted from the tissue. The quantity of protein in the samples was determined using a BCA protein assay kit (Beyotime, China), according to the manufacturer’s instructions. Samples (30 μg per lane) were separated by 12% sodium dodecyl sulfate polyacrylamide gel electrophoresis (SDS-PAGE) and transferred to polyvinylidene fluoride (PVDF) membranes. Membranes were blocked with 5% defatted milk for 2 h at room temperature (temperature of 20–25°C) and, then incubated overnight at 4°C with primary antibodies: rabbit anti-HMGB1 (1:1000 dilution, Abcam, ab18256), rabbit anti-Iba1 (1:100 dilution, Abcam, ab178847), and rabbit anti-β-actin (1:2000 dilution, Proteintech, 14395-1-AP). After three washes in PBST (0.01M PBS containing 0.1% Tween-20), the membranes were incubated with secondary antibodies (goat anti-rabbit IgG, IRDye^®^ 800CW Conjugated, 1:5000 dilution) at room temperature for 2 h. Finally, visualization of the blotted protein bands was accomplished using an infrared laser imaging system (Odyssey CLx, LI-COR, United States) and was quantified by densitometry. The relative expression levels of protein were normalized by the ratio of target protein (HMGB1 and Iba-1) to β-actin.

### Enzyme-Linked Immunosorbent Assay (ELISA)

Under anesthesia, whole blood samples were collected from the left ventricles of the rats before transcardial perfusion. Subsequently, samples were centrifuged at 3000 rpm for 10 min at room temperature. The supernatants were collected and frozen at −80°C for further analyses. Before analysis, the samples were centrifuged again, and the supernatant was used for the ELISA assay. HMGB1 concentration was determined using an HMGB1 ELISA kit (Chondrex, United States, 6160), following the manufacturer’s protocol.

For the *in vitro* experiment, the culture supernatant from HAPI microglia was collected for the ELISA. The concentrations of TNF-α, IL-1β, and IL-10 were determined using the following ELISA kits: TNF-α (Thermo Fisher, United States, 88-7340-22), IL-1β (R&D Systems, United States, DY501-05), and IL-10 (Abcam, United States, ab218796). All measurements were performed following the manufacturers’ protocols.

### Immunofluorescence Staining

Animals were anesthetized and, transcardially perfused with 0.01M PBS and 4% paraformaldehyde (PFA). The brains were then removed and post-fixed in 4% PFA immediately. After dehydration with a sucrose gradient, 20 serial coronal sections were cut across the middle of hemisphere. Sections were then washed three times with 0.01M PBS, blocked with 5% bovine serum albumin (BSA), and used for active HMGB1, Iba1, neuronal nuclei (NeuN), glial fibrillary acidic protein (GFAP), CD86, and CD206 staining. The sections were then incubated overnight at 4°C with the following primary antibodies: rabbit anti-Iba1 (1:100 dilution, Abcam, ab178847), rabbit anti-NeuN (1:300 dilution, Abcam, ab177487), mouse anti-HMGB1 (1:500 dilution, Gene Tex, GT348), mouse anti-GFAP (1:300 dilution, Cell Signaling, 3670T), rabbit anti-HMGB1 (1:1000 dilution, Abcam, ab18256), rabbit anti-CD86 (1:200 dilution, Proteintech, 13395-1-AP), rabbit anti-CD206 (1:500 dilution, Abcam, ab125028), and mouse anti- Iba1 (1:500 dilution, Abcam, ab15690). After three washes in 0.01M PBS, the sections were incubated with Cy3-conjugated goat anti-rabbit IgG (1:2000 dilution, Boster Biological Technology, BA1032) or FITC-conjugated goat anti-mouse IgG (1:2000 dilution, Boster Biological Technology, BA1101) for 1 h at room temperature. After three washes in 0.01M PBS, these were then covered with diamidino-2-phenylindole (DAPI, 1:1000, Beyotime, C1002) for 5 min. For each staining, five non-overlapping digital microscopic images of cortical areas were randomly captured using a fluorescence microscope (IX71, OLYMPUS, Japan). The number of positive cells was determined using Image-Pro Plus 6.0 (Media Cybernetics, United States).

The cultured cortical neurons were fixed with 4% PFA for 24 h after culturing in microglial-CM. They were then incubated with rabbit anti-MAP2 antibodies (1:200 dilution, Proteintech, 17490-1-AP) overnight at 4°C. After three washes in 0.01M PBS, they were incubated with Cy3-conjugated goat anti-rabbit IgG (1:2000 dilution, Boster Biological Technology, BA1032) and then covered with DAPI. Digital microscopic images were taken using the aforementioned IX71 fluorescence microscope.

### Reverse Transcription and qRT-PCR

Under deep anesthesia, the brains of the rats were removed and the cerebral cortex was rapidly separated and snap-frozen in liquid nitrogen. Total RNA was isolated using TRIzol reagent (Invitrogen, United States). The first-strand cDNA was synthesized using the Reverse Transcription System (Toyobo, Osaka, Japan), according to the manufacturer’s protocol. The relative expression level of mRNA was then assessed using the SYBR Green Real-time PCR Master Mix Kit (Toyobo, Osaka, Japan) and quantified using the Mastercycler^®^ ep realplex qRT-PCR system (Eppendorf, Germany) with glyceraldehyde 3-phosphate dehydrogenase (GAPDH) as the reference gene. All primers used in the qRT-PCR reactions were purchased from Sangon Biotech (China). The sequences of the primer pairs are described as follows: TNF-α (forward: 5′-GCA TGA TCC GAG ATG TGG AAC TGG-3′; reverse: 5′-CGC CAC GAG CAG GAA TGA GAA G-3′); IL-6 (forward: 5′-AGG AGT GGC TAA GGA CCA AGA CC-3′; reverse: 5′-TGC CGA GTA GAC CTC ATA GTG ACC-3′); IL-1β (forward: 5′-ATC TCA CAG CAT CTC GAC AAG-3′; reverse: 5′-CAC ACT AGC AGG TCG TCA TCC-3′); iNOS (forward: 5′-AGA TCC CGA AAC GCT ACA CTT-3′; reverse: 5′-TGC GGC TGG ACT TCT CAC TC-3′); arginase-1 (forward: 5′-ACA TCA ACA CTC CGC TGA CAA CC-3′; reverse: 5′-GCC GAT GTA CAC GAT GTC CTT GG-3′); TGF-β (forward: 5′-GGC ACC ATC CAT GAC ATG AAC CG-3′; reverse: 5′-GCC GTA CAC AGC AGT TCT CTG-3′); IL-4 (forward: 5′-CAA GGA ACA CCA CGG AGA ACG AG-3′; reverse: 5′-CTT CAA GCA CGG AGG TAC ATC ACG-3′); GAPDH (forward: 5′-GAC ATG CCG CCT GGA GAA AC-3′; reverse: 5′-AGC CCA GGA TGC CCT TTA GT-3′). The relative expression of mRNA was calculated using the 2^–ΔΔCt^ method.

### Infarct Ratio Calculation

The rat brains were rapidly removed 3 days post-HI and frozen in a freezer at −20°C for 20 min. They were then taken out and four 1.5 mm thick sections were sliced. These sections were placed in 2% TTC staining solution, and incubated at 37°C for 20 min in the dark. Finally, the stained brain slices were placed on a drape for photographing. The aforementioned Image-Pro Plus 6.0 software was used to calculate the volume of each section. The following formula was used to calculate infarct ratio:

$$R\,a\,t\,i\,o\,o\,f\,i\,n\,f\,a\,r\,c\,t=\frac{i\,n\,f\,a\,r\,c\,t\,v\,o\,l\,u\,m\,e}{t\,o\,t\,a\,l\,s\,e\,c\,t\,i\,o\,n\,v\,o\,l\,u\,m\,e}\times 100$$

### Brain Water Content

Rats were sacrificed under anesthesia24 h after HI. The brains were bisected to generate two hemispheres (ipsilateral and contralateral to the injury) which were then immediately weighed (wet weight). The hemispheres were then put in an oven (105°C) for 48 h and weighed again (dry weight). The following formula was used to calculate brain water content:

$$Brainwatercontent\left(\%\right)=\left[\frac{w\,e\,t\,w\,e\,i\,g\,h\,t-d\,r\,y\,w\,e\,i\,g\,h\,t}{w\,e\,t\,w\,e\,i\,g\,h\,t}\right]\times 100$$

### Statistical Analysis

All data are shown as means ± SEM. Data from the different experimental groups were analyzed using one-way ANOVA and the Tukey test for *post hoc* comparisons. Statistical Package for the Social Sciences 19.0 (SPSS, IBM, United States) and GraphPad Prism 5.0 (GraphPad, San Diego, CA, United States) were used for this statistical analysis. A *p*-value < 0.05 was considered statistically significant.

## Results

### HMGB1 Was Upregulated in Both the Cerebral Cortex and Serum After HI

**FIGURE 1:**
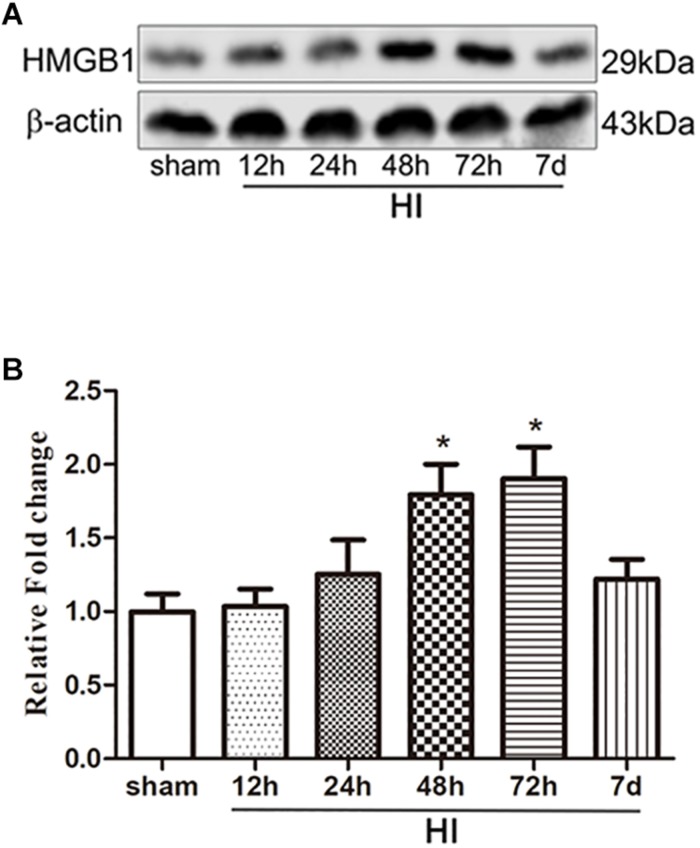
Expression of HMGB1 in the cerebral cortex after HI. **(A)** Western blot bands of HMGB1 expression in the cerebral cortex of P7 SD rats at different time points from 12 h to 7 days after HI. **(B)** Quantitative analyses of the western blot bands (relative OD value) at different time points from 12 h to 7 days after HI, comparing to the sham group (*n* = 5 for each group). The expression of HMGB1 dramatically increased 24 h after HI and peaked 72 h after HI. Bars represent the mean ± SEM. *^∗^P* < 0.05 compared to the sham group.

**FIGURE 2:**
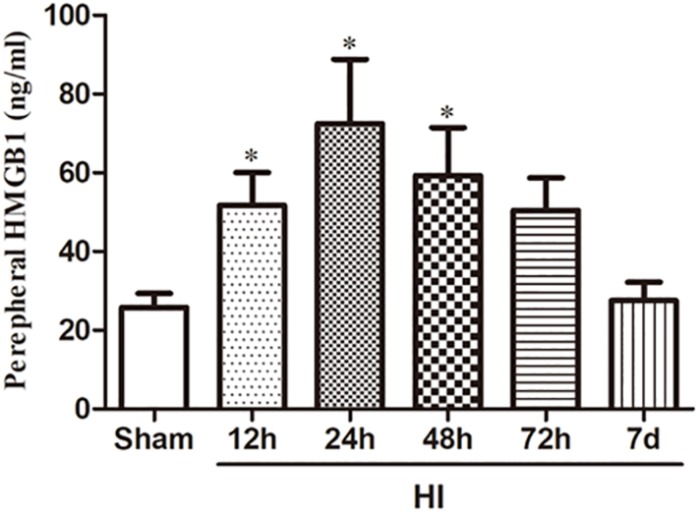
Expression of HMGB1 in serum after HI. HMGB1 level in the peripheral blood detected by ELISA was increased significantly 12, 24, and 48 h after HI exposure with a peak level 24 h after HI comparing to the sham group (*n* = 10 for each group except *n* = 9 for the 12 and 24 h group). The serum level of HMGB1 7 days after HI was decreased to a similar level of the sham group. Bars represent the mean ± SEM. *^∗^P* < 0.05 compared to the sham group.

To investigate the effect of HI on HMGB1, we examined the expression of HMGB1 in the ipsilateral cerebral cortex and peripheral blood by western blotting and ELISA, respectively. Western blot analysis revealed that the expression of HMGB1 was increased in the ipsilateral cerebral cortex 48 h after HI, peaked 72 h after HI, and subsequently decreased to baseline level compared to the sham group (both *p* < 0.05) (Figures 1A,B). ELISA results revealed that the expression of HMGB1 was rapidly up-regulated in serum after HI, peaking at 24 h and gradually returning to a normal level (51.72 ± 8.32, 72.43 ± 16.38, 59.32 ± 12.18 vs. 25.78 ± 3.58 ng/mL at 12, 24, 48 h post-HI compared to sham group respectively; all *p* < 0.05) (Figure 2).

### Cortical HMGB1 Expression Was Increased in Microglia, but Not in Neurons or Astrocytes

**FIGURE 3:**
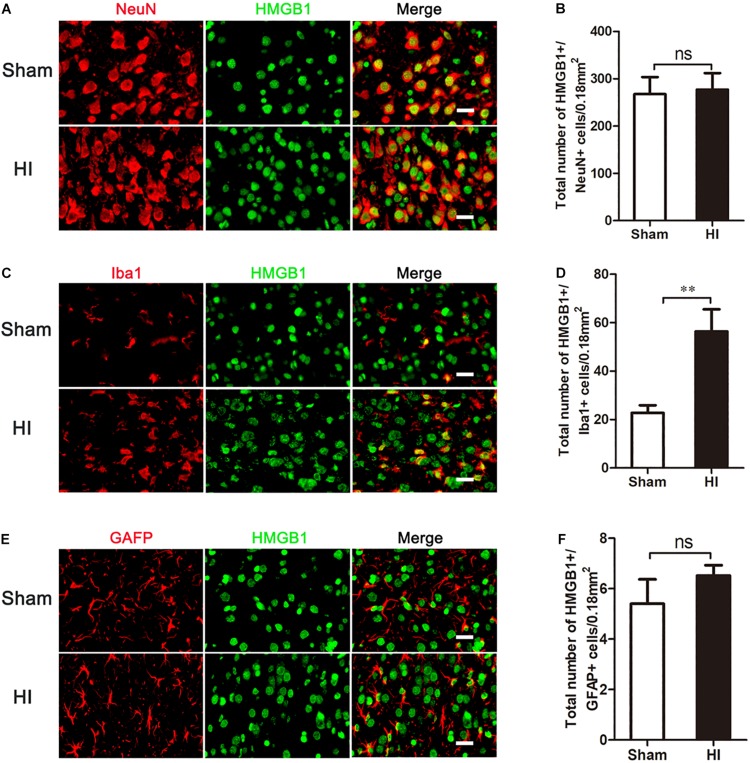
Co-expression of HMGB1 and neural cells in the cerebral cortex after HI. **(A,C,E)** Double immunofluorescent labeling for HMGB1 with NeuN **(A)**, Iba-1 **(C)** and GFAP **(E)** in the cerebral cortex 72 h after HI. Scale bars = 20 μm. NeuN, Iba-1 and GFAP positive cells are red, HMGB1 positive cells are green and merged cells are yellow. **(B,D,F)** Statistical results of NeuN+/HMGB1+, Iba-1+/HMGB1+ and GFAP+/HMGB1+ cell numbers in 0.18 mm^2^ area (*n* = 6 for each group). Iba-1+/HMGB1+ cell numbers significantly increased after HI. There were no significant changes in NeuN+/HMGB1+ and GFAP+/HMGB1+ cell numbers after HI compared to the sham group. Bars represent the mean ± SEM. *^∗∗^P* < 0.01, ns, no significance compared to the sham group.

As HMGB1 is widely expressed in the brain, we then explored in which type of cells HMGB1 was up-regulated. Double-labeled immunofluorescence of HMGB1 was performed in neurons, microglia, and astrocytes. In the sham group, cortical HMGB1 was primarily expressed in neurons (NeuN+/HMGB1+) (267.40 ± 36.04/0.18 mm^2^), with low expression in microglia (Iba1+/HMGB1+) (22.72 ± 3.17/0.18 mm^2^) and astrocytes (GFAP+/HMGB1+) (5.40 ± 0.97/0.18 mm^2^) (Figures 3A,C,E). However, 72 h after HI, HMGB1 was increased in microglia [56.36 ± 9.13/0.18 mm^2^ (HI) vs. 22.72 ± 3.17/0.18 mm^2^ (Sham), *p* < 0.01], but not in neurons [267.40 ± 36.04/0.18 mm^2^ (HI) vs. 276.90 ± 34.98/0.18 mm^2^ (Sham), *p* > 0.05] or astrocytes [(5.40 ± 0.97/0.18 mm^2^ (HI) vs. 6.52 ± 0.41/0.18 mm^2^ (Sham), *p* > 0.05] (Figures 3B,D,F).

### Microglial Activation Occurred in the Cerebral Cortex After HI

**FIGURE 4:**
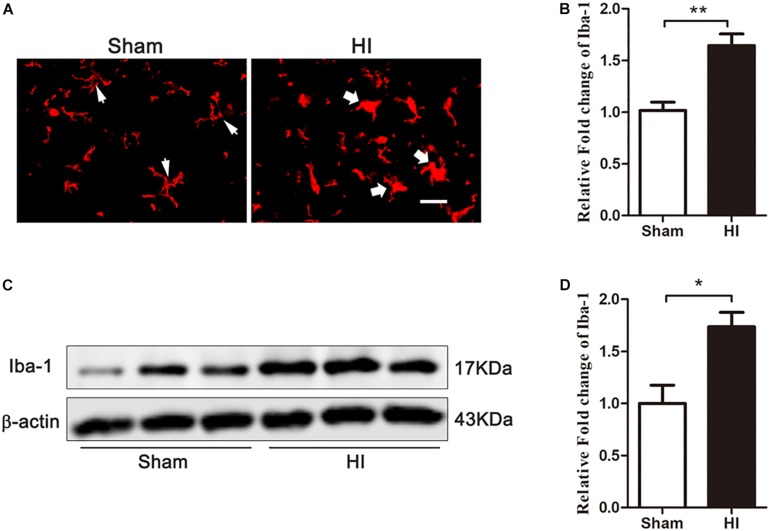
Expression of microglia in the cerebral cortex after HI. **(A)** Immunofluorescent labeling of microglia (Iba-1) in the cerebral cortex 72 h after HI. The morphology of microglia changed from branch in sham group (white narrow arrows) to ameba-like in HI group (white wide arrows) after HI exposure. Scale bar = 20 μm. **(B)** Quantitative analyses of Iba-1 fluorescence intensity (relative OD value) showed that there was an increased expression of Iba-1 (*n* = 6 for each group). **(C)** The expression of Iba-1 in the cerebral cortex detected by western blot 72 h after HI. **(D)** Quantitative analyses of the western blot bands (relative OD value) revealed that there was a significantly increase in the expression of Iba-1 (*n* = 6 for each group). Bars represent the mean ± SEM. *^∗^P* < 0.05 compared to the sham group, *^∗∗^P* < 0.01 compared to the sham group.

Previous studies have shown that microglia are activated in the pathophysiology of HIBD (Ferrazzano et al., 2013; Cengiz et al., 2019), and HMGB1, a pro-inflammatory factor, is upregulated in these activated microglia. Therefore, we explored whether microglial activation was induced in the cerebral cortex after HI. Immunofluorescence (Figures 4A,B) and western blot (Figures 4C,D) experiments showed that 72 h after HI injury, the expression of Iba-1, a marker of microglia, was significantly higher in the HI group than in the sham group (*p* < 0.01 and, *p* < 0.05, respectively). Meanwhile, microglial morphology changed from branch to amoeba-like after HI exposure. These changes demonstrated that cortical microglia were activated 72 h after HI.

### HMGB1 Inhibition Altered M1/M2 Microglial Polarization and Cytokine Transcription

**FIGURE 5:**
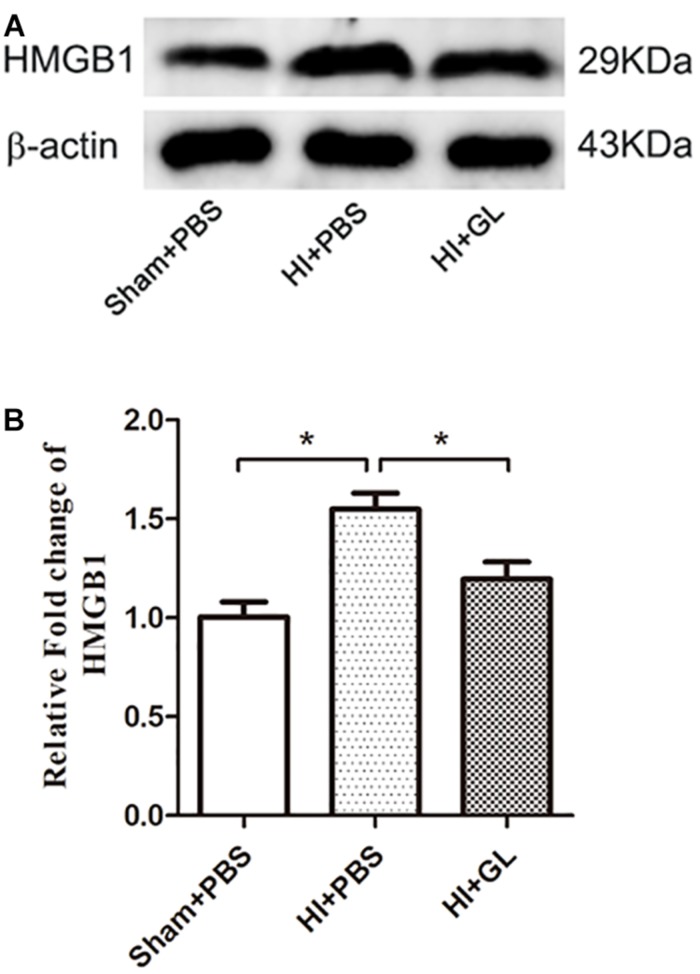
Effect of GL on the expression of HMGB1 in the cerebral cortex after HI. **(A)** Western blot bands of HMGB1 expression in the cerebral cortex 72 h after HI. **(B)** Quantitative analyses of the western blot bands (relative OD value) showed that GL effectively inhibited the expression of HMGB1 in the cerebral cortex 72 h after HI (*n* = 5 for each group). Bars represent the mean ± SEM. *^∗^P* < 0.05.

**FIGURE 6:**
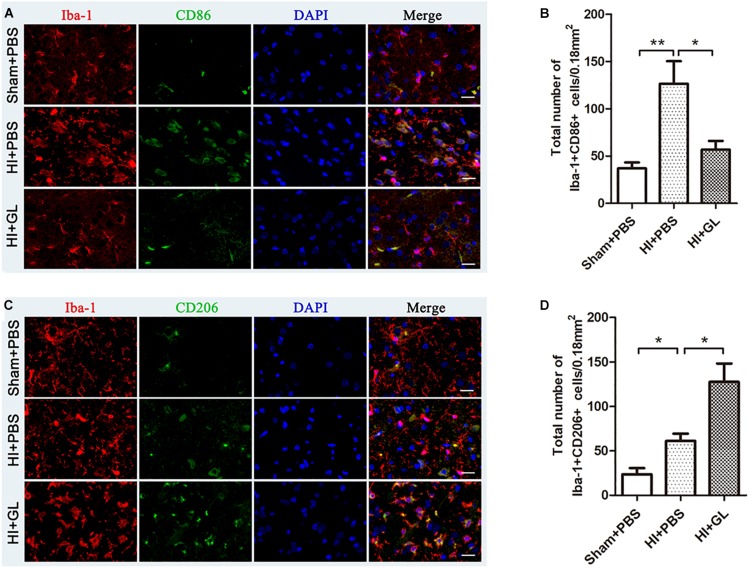
Effect of GL on M1 and M2 phenotypes of microglia in the cerebral cortex after HI. **(A)** Immunofluorescent labeling of M1 phenotype (Iba-1+/CD86+) in the cerebral cortex 72 h after HI. Scale bars = 20 μm, Iba-1 positive cells are red, CD86 positive cells are green, DAPI-stained nuclei are blue and merged cells are yellow. **(B)** The statistical results of Iba-1+/CD86+ cell numbers in 0.18 mm^2^ area. The expression of M1 phenotype significantly increased after HI. While it had a decrease in HI + GL group compared to HI + PBS group. **(C)** Immunofluorescent labeling of M2 phenotype (Iba-1+/CD206+) in the cerebral cortex 72 h after HI. Scale bars = 20 μm, Iba-1 positive cells are red, CD206 positive cells are green, DAPI-stained nuclei are blue and merged cells are yellow. **(D)** The statistical results of Iba-1+/CD206+ cell numbers in 0.18 mm^2^ area. The expression of M2 phenotype significantly increased after HI and it had an increase in HI + GL group compared to HI + PBS group. *N* = 6 for each group, bars represent the mean ± SEM. *^∗^P* < 0.05, *^∗∗^P* < 0.01.

Having observed microglial activation, we further analyzed the M1/M2 polarization of microglia in the cerebral cortex and investigated if this was affected by GL, a specific inhibitor of HMGB1. We first used western blotting to detect whether the expression of HMGB1 in the cerebral cortex could be inhibited by GL pretreatment (Figures 5A,B). It was showed that GL could effectively attenuate the expression of HMGB1 in the cerebral cortex 72 h after HI. Then Iba1+/CD86+ and Iba1+/CD206+ co-staining were used to identify M1 and M2 phenotypes, respectively (Figures 6A–D). The results showed that the expression of both M1 and M2 phenotypes was significantly increased 72 h after HI [37.00 ± 6.29 cells/0.18mm^2^ (Sham + PBS) vs. 126.60 ± 23.74 cells/0.18mm^2^ (HI + PBS); 23.60 ± 6.94 cells/0.18mm^2^ (Sham + PBS) vs. 61.20 ± 8.12 cells/0.18mm^2^ (HI + PBS), *p* < 0.01 and *p* < 0.05, respectively]. When rats were pretreated with GL, the number of M1 microglia (Iba1+/CD86+ cells) significantly reduced [126.60 ± 23.74 cells/0.18 mm^2^ (HI + PBS) vs. 56.80 ± 9.12 cells/0.18 mm^2^ (HI + GL), *p* < 0.05] (Figures 6A,B), while the number of M2 microglia (Iba1+/CD206+ cells) significantly increased [61.20 ± 8.12 cells/0.18 mm^2^ (HI + PBS) vs. 127.60 ± 20.60 cells/0.18 mm^2^ (HI + GL), *p* < 0.05] (Figures 6C,D).

**FIGURE 7:**
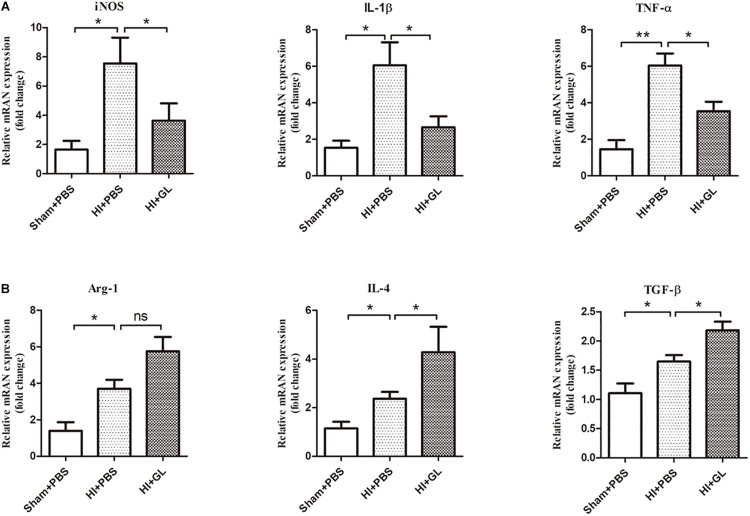
Effect of GL on M1 and M2 functional cytokines in the cerebral cortex after HI. **(A)** The mRNA levels of M1 functional cytokines (iNOS, IL-1β, and TNF-α) were detected using qRT-PCR 72 h after HI. Results showed that the mRNA expression of M1 functional cytokines all increased after HI. While there was a significant decrease in HI + GL group compared to HI + PBS group. **(B)** The mRNA levels of M2 functional cytokines (Arg-1, IL-4, and TGF-β) were detected using qRT-PCR 72h after HI. Results showed that the expression of M2 functional cytokines all increased after HI and there was a higher level in HI + GL group compared to HI + PBS group. *N* = 6 for each group, bars represent the mean ± SEM, *^∗^P* < 0.05, *^∗∗^P* < 0.01, ns, no significance.

We then detected the mRNA expression of M1 and M2 inflammatory factors using qRT-PCR. Results showed that the mRNA expression of M1 and M2 functional cytokines was significantly increased in the HI + PBS compared to the sham + PBS group 72 h after HI (*p* < 0.05, Figures 7A,B). The mRNA expression of M1 functional cytokines (iNOS, TNF-α, and IL-1β) was significantly decreased in the HI + GL group compared to the HI + PBS group (all *p* < 0.05, Figure 7A). In contrast, the mRNA expression of M2 functional cytokines (arginase1, IL-4, and TGF-β) was significantly increased in the HI + GL group compared to the HI + PBS group (all *p* < *0.05* except for arginase1, Figure 7B).

### HMGB1 Aggravated Microglial-Induced Neurotoxicity *in vitro*

**FIGURE 8:**
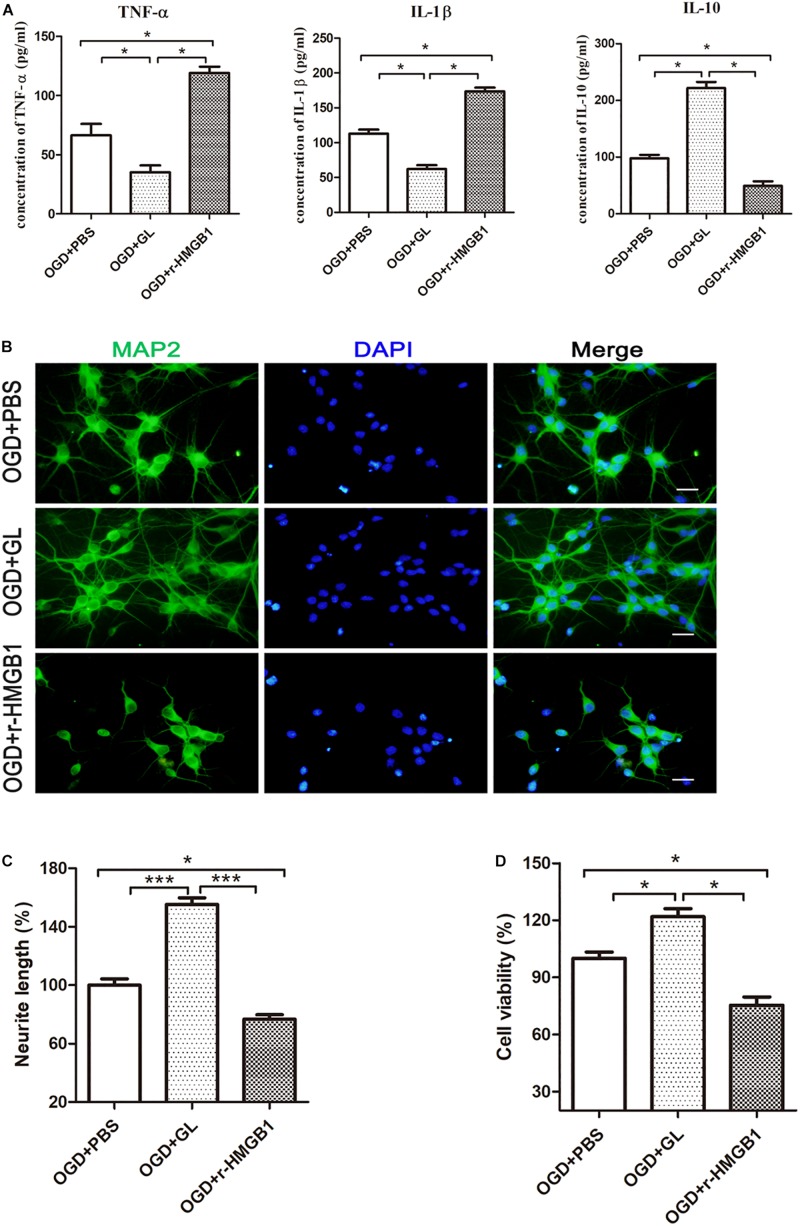
Effect of HMGB1 on microglia-induced neurotoxicity *in vitro*. **(A)** HAPI microglia was pretreated with r-HMGB1 or GL and then undergoing OGD for 24 h. The expression of TNF-α, IL-1β and IL-10 in culture supernatant was measured by ELISA (*n* = 4 wells per group). **(B)** Immunofluorescent staining of primary cortical neurons (MAP2, green) with DAPI (blue) 48 h after incubation with different CM from HAPI microglial medium. Scale bars = 20 μm. **(C)** Quantitative analyses of relative neurite length of primary neurons after immunofluorescent staining using ImageJ. *N* = 4 wells for each group, 3 digital microscopic images for each well. **(D)** Cell viability of primary neurons 48 h after incubation with different CM from HAPI microglial medium using CCK8 method (*n* = 4 wells for each group). Bars represent the mean ± SEM, *^∗^P* < 0.05, *^∗∗∗^P* < 0.001.

To further elucidate the effect of HMGB1 on microglial phenotype, an OGD model of HAPI microglial cells was established *in vitro.* The effects of r-HMGB1 and its inhibitor GL, on the phenotypic changes of HAPI microglial cells were examined (Figure 8A). ELISA analysis showed that GL significantly reduced the expression of the M1-associated inflammatory cytokines TNF-α [66.44 ± 4.84 pg/mL (OGD + PBS) vs. 35.08 ± 2.94 pg/mL (OGD + GL), *p* < 0.05] and IL-1β [113.00 ± 2.99 pg/mL (OGD + PBS) vs. 62.21 ± 2.85 pg/mL (OGD + GL), *p* < 0.05], and significantly increased the expression of the M2-associated inflammatory cytokine IL-10 [97.89 ± 3.08 pg/mL (OGD + PBS) vs. 221.90 ± 5.43 pg/mL (OGD + GL), *p* < 0.05]. In contrast, r-HMGB1 significantly increased the expression of TNF-α [66.44 ± 4.84 pg/mL (OGD + PBS) vs. 119.00 ± 2.70 pg/mL (OGD + r-HMGB1), *p* < 0.05] and IL-1β [113.00 ± 2.99 pg/mL (OGD + PBS) vs. 173.60 ± 2.77 pg/mL (OGD + r-HMGB1), *p* < 0.05], and significantly reduced the expression of IL-10 [97.89 ± 3.08 pg/mL (OGD + PBS) vs. 49.37 ± 3.98 pg/mL (OGD + GL), *p* < 0.05]. These results indicated that under OGD conditions, HMGB1 promoted the polarization of microglia to a M1-like phenotype. In contrast, inhibition of HMGB1 promoted the polarization of microglia to a M2-like phenotype.

Next, the effect of different CM from HAPI microglial cells on neurons was examined (Figures 8B–D). We defined CM from M1 and M2-like phenotypes, as M1-CM and M2-CM, respectively. We first used MAP2 as a marker to explore the effects of different CMs on the length of nerve dendrites using an immunofluorescence assay. It was noted that the mean neurite length of MAP2-positive cells significantly decreased in the presence of M1-CM. In contrast, the mean neurite length of MAP2-positive cells significantly increased in the presence of M2-CM (Figures 8B,C). The survival of cortical neurons was then evaluated using a CCK-8 assay. This showed that M1-CM from the OGD+ r-HMGB1 group significantly reduced the cell viability of the primary cortical neurons, while M2-CM from the OGD + GL group significantly increased the cell viability of the primary cortical neurons, compared to the OGD + PBS control group (Figure 8D). These results suggested that HMGB1 could aggravate microglial-induced neurotoxicity in OGD conditions.

### HMGB1 Inhibition Alleviated HI-Induced Brain Injury

**FIGURE 9:**
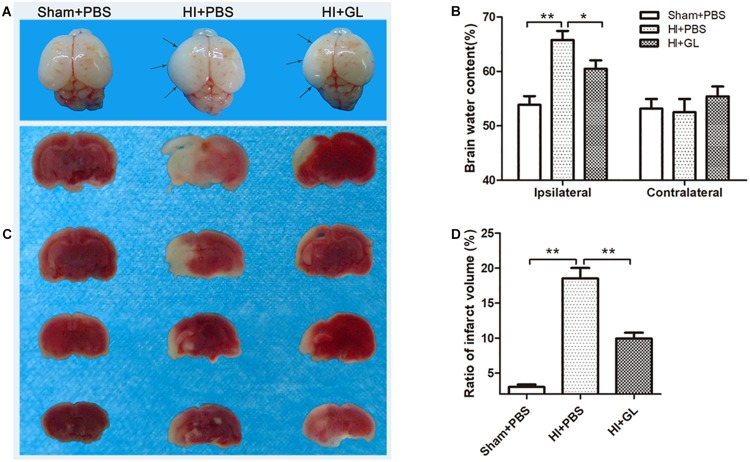
Effect of GL treatment on HI-induced brain injury. **(A)** Whole brain photos showing cerebral edema 24 h after HI. Gray arrows indicate sites of significant edema. Cerebral edema area in HI + GL group was smaller than HI + PBS group. **(B)** The brain water content measurement showed that the ipsilateral brain water content in HI + GL group was significantly lower compared to the HI + PBS group (*n* = 6 for each group). **(C)** TTC staining of all treatment groups 72 h after HI. Normal tissues were red or pink, while infract tissues were white. The white area in HI + GL group was smaller than HI + PBS group. **(D)** Statistical results showed that the ratio of infract volume in HI + GL group was significantly lower compared to HI + PBS group (*n* = 5 for each group). Bars represent the mean ± SEM, *^∗^P* < 0.05, *^∗∗^P* < 0.01.

As HMGB1 induced microglial polarization to an M1 phenotype, leading to increased neurotoxicity, we then explored whether inhibition of HMGB1 could attenuate HI-induced brain damage using edema assessment and cerebral infarction detection (Figures 9A–D). As shown in Figure 9A, the ipsilateral side of the brain was visibly edematous 72 h after HI insult. Pretreatment with GL alleviated this edema and morphological damage in the HI group. As shown in Figure 9B, the water content of the ipsilateral hemispheres was significantly increased in the HI group compared to the sham group. The inhibition of HMGB1 significantly alleviated the water content of the ipsilateral hemispheres in the HI group (*p* < 0.05). TTC staining showed that HI insult substantially increased ipsilateral infarct size, which was reversed by pretreatment with HMGB1 inhibition (*p* < 0.05, Figures 9C,D). These results suggested that the inhibition of HMGB1 could alleviate HI-induced brain damage.

## Discussion

The present study investigated whether HMGB1 has a role in the pathogenesis of neonatal rat HIBD, focusing on the polarization of microglia. It was found that HMGB1 led to an imbalance in M1/M2 microglial polarization in the cortex, thus aggravating brain damage. This was alleviated by inhibiting HMGB1 with GL in HIBD.

HMGB1, a damage-associated protein, mediates neuroinflammation and brain damage in many neurological diseases including ischemic stroke (Angelopoulou et al., 2018; Paudel et al., 2018; Ye et al., 2019). Previous studies demonstrated that HMGB1 increased early at 3 h in serum and decreased in cerebral cortex after HI (Chen et al., 2019). Similarly, we found that HMGB1 was upregulated in peripheral blood after HI insult. But in our study, ipsilateral cortical HMGB1 also increased and serum HMGB1 increased 12 h after HI, inconsistent with the aforementioned results. These different results may be due to the different temperatures and different severity of injury after suffering from HI. It is suggested that pathological change and cerebral metabolic rate are different under different temperatures, and different temperatures can even affect clinical efficacy in hypothermia-treated HIE (Kim et al., 2014). And due to individual variation in animals, different severity of brain injury may occur, which could also lead to the discrepancy above.

Besides the serum and cortical upregulation of HMGB1, we also found that the expression of HMGB1 increased in peripheral blood much earlier than in the brain. There are two potential mechanisms underlying this experimental phenomenon. One possibility is that the early increase of peripheral HMGB1 comes from brain tissue damage. As a typical damage-associated protein, HMGB1 could be released from damaged or necrotic brain tissue after HI insult, directing blood-brain barrier (BBB) breakdown and entering peripheral blood immediately, leading to a rapid increase of peripheral HMGB1 (Faraco et al., 2007; Ye et al., 2019). In addition, stimulation such as surgery and HI insult may have induced the release of HMGB1 from peripheral organs, causing peripheral HMGB1 to increase rapidly (Jellema et al., 2013; Terrando et al., 2016). The increased peripheral HMGB1 could in turn damage BBB and enter the brain, along with HMGB1 from the damaged brain tissue to lead to an increase of cerebral HMGB1. Our previous study showed that serum HMGB1 levels were significantly elevated in persistent pulmonary hypertension of the newborn (PPHN) and decreased dramatically after PPHN resolution (Tang et al., 2019). A meta-analysis by Le et al. (2018) demonstrated that circulating blood HMGB1 levels increased in ischemic stroke, with a higher HMGB1 level indicating a more serious condition. The rapid rise observed in peripheral HMGB1 after HI suggested that HMGB1 is sensitive to HI insult, and may potentially be useful as a biomarker in the early stage of HIBD.

In our study, we found that HMGB1 was widely expressed in the neurons of neonatal rat brains. However, the upregulation of HMGB1 after HI was characterized by an increased expression in microglia, but not in neurons or astrocytes. Several studies have demonstrated that HMGB1 can be transferred from the nucleus of the neuron to the cytoplasm or released extracellularly by stimulation such as brain ischemia (Qiu et al., 2008; Zhang et al., 2016; Chen et al., 2019) and subarachnoid hemorrhage (Sun et al., 2014). Under severe cerebral ischemic conditions, neuronal cells undergo apoptosis or necrosis by the induction of a number of pathways (Thal et al., 2011). As a result, HMGB1 could be released from necrotic neurons and interact with microglia as an extracellular cytokine (Frasch and Nygard, 2017). Based on the above research, it is therefore likely that HMGB1 was transferred extracellularly from neuronal nuclei or released from necrotic neurons after HI insult. This neuronal HMGB1 could then serve as an extracellular factor, binding to microglia and, resulting in the increase of HMGB1+/Iba1+ cells. As a group of innate immune cells in the central nervous system, it is known that microglial-mediated neuroinflammation plays an important role in the pathogenesis of many neurological diseases, including HIBD (Fonken et al., 2016; Zhang et al., 2016; Kigerl et al., 2018). As an important inflammatory factor, the binding of HMGB1 to microglia could further promote this neuroinflammation (Sun et al., 2018), in turn triggering an inflammation cascade response and, aggravating brain damage. Further, the *in vitro* experiments in our study showed that HMGB1 increased the neurotoxicity of neurons under the condition of OGD (Figure 8). It is implied that the increase of HMGB1+/Iba1+ cells may result in a decrease of HMGB1+/NeuN+ cells. However, we did not find a decrease in the number of HMGB1+/NeuN+ cells 72 h after HI. Due to the sustained neuroinflammation, there is a great possibility that the number of HMGB1+/NeuN+ cells will decrease after 72 h post HI. Thus, inhibition of HMGB1 expression may help to reduce neuroinflammation.

Studies have shown that microglia can be activated after HI stimulation (Ohsawa and Kohsaka, 2011; Serdar et al., 2019). In our study, HI insult resulted in an increased expression of Iba-1 and a change in microglial morphology from branch to ameba-like. These results indicated that cortical microglia were activated after HI, consistent with previous research (Harry, 2013; Cengiz et al., 2019). Microglia can be differentiated into different subtypes after activation. The two main subtypes are M1 and M2 microglia, characterized by pro-inflammatory (neurotoxic) and anti- inflammatory (neuroprotective) phenotypes, respectively (Olah et al., 2011; Patel et al., 2013). In this study, the HMGB1 inhibitor GL was used to investigate the effect of HMGB1 on the M1/M2 polarization of microglia during the pathogenesis of HIBD. The results showed that the expression of both M1 and M2 microglia was significantly increased 72 h after HI. Inhibition of HMGB1 by GL reduced the expression of the M1 phenotype and promoted the expression of the M2 phenotype, indicating that HMGB1 was involved in the imbalance of M1/M2 microglial polarization in HIBD.

In addition, an *in vitro* experiment was conducted to mimic the HI process *in vivo*. We investigated the polarization state of microglia and the effect of microglia on HMGB1 expression in neurons during the process of OGD. We found that HMGB1 promoted the polarization of microglia to an M1 phenotype, enhancing the expression of pro-inflammatory factors such as TNF-α and IL-1β, and resulting in a decrease in neuronal activity and dendritic length. However, the above phenomenon could be neutralized by inhibiting HMGB1 with GL. We conducted further animal experiments and found that HMGB1 inhibition could reduce brain edema and cerebral infarction area after HI. The above effects of HMGB1 are similar to those reported by Gao et al. (2018) and Sun et al. (2018). Taken together, these findings suggest that HMGB1 may cause neuroinflammation and neuronal damage by inducing an imbalance of M1/M2 microglial polarization, leading to brain damage.

There exists a number of limitations of our study. As different pathological changes occur at different time points after HI (Park et al., 2018), the window of time during which post-HI treatment is administered has a significant impact on prognosis. Unfortunately, in our study, GL was only administered as a pre-treatment before HI and not at different time points after HI. In addition, this study did not explore the underlying mechanisms and specific pathways by which HMGB1 regulates the M1/M2 polarization of microglia. Finally, this study only examined the acute phase of HI (0–72 h), whereas it is known that, perinatal HI has a long-term effect on the development of the nervous system in children (Papazian, 2018; Sanches et al., 2019). This study did not investigate the effects of HMGB1 on the development of the brain in the late phase of HI. The aforementioned points therefore need to be addressed in further research.

## Conclusion

Our findings suggest that HMGB1 may lead to an imbalance of M1/M2 microglial polarization in the cortex and thus neuronal injury. Such findings suggest that pharmacological blockade of HMGB1 signaling may attenuate this imbalanced polarization of microglia and thus could be used as a therapeutic strategy against brain injury in HIBD.

## Data Availability Statement

The raw data supporting the conclusions of this article will be made available by the authors, without undue reservation, to any qualified researcher.

## Ethics Statement

All experiments were performed in accordance with the guidelines for experimental animal use of Central South University. The protocol was approved by the Ethics Committee of the Third Xiangya Hospital of Central South University (No. 2016-S006).

## Author Contributions

YS, ZF, and ZT performed the experiments, analyzed the data, and wrote the manuscript. NH and BW performed the experiments and analyzed the data. YS and MH designed the study and revised the manuscript. All of the authors read and approved the final manuscript.

## Conflict of Interest

The authors declare that the research was conducted in the absence of any commercial or financial relationships that could be construed as a potential conflict of interest.

## References

[B1] Andersson U. Yang H. Harris H. (2018). High-mobility group box 1 protein (HMGB1) operates as an alarmin outside as well as inside cells. *Semin. Immunol.* 38 40–48. 10.1016/j.smim.2018.02.011 29530410

[B2] Angelopoulou E. Piperi C. Papavassiliou A. G. (2018). High-mobility group box 1 in Parkinson’s disease: from pathogenesis to therapeutic approaches. *J. Neurochem.* 146 211–218. 10.1111/jnc.14450 29676481

[B3] Barkhuizen M. van den Hove D. L. Vles J. S. Steinbusch H. W. Kramer B. W. Gavilanes A. W. (2017). 25 years of research on global asphyxia in the immature rat brain. *Neurosci. Biobehav. Rev.* 75 166–182. 10.1016/j.neubiorev.2017.01.042 28161509

[B4] Bhalala U. S. Koehler R. C. Kannan S. (2014). Neuroinflammation and neuroimmune dysregulation after acute hypoxic-ischemic injury of developing brain. *Front, Pediatr.* 2:144. 10.3389/fped.2014.00144 25642419 PMC4294124

[B5] Cengiz P. Zafer D. Chandrashekhar J. H. Chanana V. Bogost J. Waldman A. (2019). Developmental differences in microglia morphology and gene expression during normal brain development and in response to hypoxia-ischemia. *Neurochem. Int.* 127 137–147. 10.1016/j.neuint.2018.12.016 30639264 PMC7285704

[B6] Chen X. Zhang J. Kim B. Jaitpal S. Meng S. S. (2019). High-mobility group box-1 translocation and release after hypoxic ischemic brain injury in neonatal rats. *Exp. Neurol.* 311 1–14. 10.1016/j.expneurol.2018.09.007 30217406 PMC6261802

[B7] Colton C. A. (2009). Heterogeneity of microglial activation in the innate immune response in the brain. *J. Neuroimmune. Pharmacol.* 4 399–418. 10.1007/s11481-009-9164-4 19655259 PMC2773116

[B8] Faraco G. Fossati S. Bianchi M. E. Patrone M. Pedrazzi M. (2007). High mobility group box 1 protein is released by neural cells upon different stresses and worsens ischemic neurodegeneration in vitro and in vivo. *J. Neurochem.* 103 590–603. 10.1111/j.1471-4159.2007.04788.x 17666052

[B9] Ferrazzano P. Chanana V. Uluc K. Fidan E. Akture E. (2013). Age-dependent microglial activation in immature brains after hypoxia- ischemia. *CNS Neurol. Disord. Drug Targets* 12 338–349. 10.2174/1871527311312030007 23469850 PMC3674227

[B10] Fonken L. K. Frank M. G. Kitt M. M. D’Angelo H. M. Norden D. M. (2016). The alarmin HMGB1 mediates age-induced neuroinflammatory priming. *J. Neurosci.* 36 7946–7956. 10.1523/JNEUROSCI.1161-16.2016 27466339 PMC4961779

[B11] Frasch M. G. Nygard K. L. (2017). Location, location, location: appraising the pleiotropic function of HMGB1 in fetal brain. *J. Neuropathol. Exp. Neurol.* 76 332–334. 10.1093/jnen/nlx004 28340120 PMC5965030

[B12] Gao T. Chen Z. Chen H. Yuan H. Wang Y. (2018). Inhibition of HMGB1 mediates neuroprotection of traumatic brain injury by modulating the microglia/macrophage polarization. *Biochem. Biophys. Res. Commun.* 497 430–436. 10.1016/j.bbrc.2018.02.102 29448108

[B13] Guazzi S. Strangio A. Franzi A. T. Bianchi M. E. (2003). HMGB1, an architectural chromatin protein and extracellular signalling factor, has a spatially and temporally restricted expression pattern in mouse brain. *Gene. Expr. Patterns* 3 29–33. 10.1016/s1567-133x(02)00093-5 12609598

[B14] Harry G. J. (2013). Microglia during development and aging. *Pharmacol. Ther.* 139 313–326. 10.1016/j.pharmthera.2013.04.013 23644076 PMC3737416

[B15] Jellema R. K. Lima P. V. Zwanenburg A. Ophelders D. R. De Munter S. (2013). Cerebral inflammation and mobilization of the peripheral immune system following global hypoxia-ischemia in preterm sheep. *J. Neuroinflammation* 10:13. 10.1186/1742-2094-10-13 23347579 PMC3614445

[B16] Jin X. Liu M. Y. Zhang D. F. Zhong X. Du K. Qian P. (2019). Natural products as a potential modulator of microglial polarization in neurodegenerative diseases. *Pharmacol. Res.* 145:104253. 10.1016/j.phrs.2019.104253 31059788

[B17] Kaur C. Rathnasamy G. Ling E. A. (2017). Biology of microglia in the developing brain. *J. Neuropathol. Exp. Neurol.* 76 736–753. 10.1093/jnen/nlx056 28859332

[B18] Kigerl K. A. Lai W. Wallace L. M. Yang H. Popovich P. G. (2018). High mobility group box-1 (HMGB1) is increased in injured mouse spinal cord and can elicit neurotoxic inflammation. *Brain Behav. Immun.* 72 22–33. 10.1016/j.bbi.2017.11.018 29175543 PMC6681463

[B19] Kim J. H. Yun S. H. Jang K. H. Park J. Han H. S. (2014). Delayed and prolonged local brain hypothermia combined with decompressive craniectomy: a novel therapeutic strategy that modulates glial dynamics. *Exp. Neurobiol.* 23 115–123. 10.5607/en.2014.23.2.115 24963275 PMC4065824

[B20] Le K. Mo S. Lu X. Idriss A. A. Yu D. Guo Y. (2018). Association of circulating blood HMGB1 levels with ischemic stroke: a systematic review and meta-analysis. *Neurol. Res.* 40 907–916. 10.1080/01616412.2018.1497254 30015578

[B21] Liu Y. Prasad R. Wilson S. H. (2010). HMGB1: roles in base excision repair and related function. *Biochim. Biophys. Acta* 1799 119–130. 10.1016/j.bbagrm.2009.11.008 20123074 PMC2818529

[B22] Merenmies J. Pihlaskari R. Laitinen J. Wartiovaara J. Rauvala H. (1991). 30-KDa heparin-binding protein of brain (amphoterin) involved in neurite outgrowth. Amino acid sequence and localization in the filopodia of the advancing plasma membrane. *J. Biol Chem.* 266 16722–16729. 1885601

[B23] Ohsawa K. Kohsaka S. (2011). Dynamic motility of microglia: purinergic modulation of microglial movement in the normal and pathological brain. *Glia* 59 1793–1799. 10.1002/glia.21238 21901756

[B24] Olah M. Biber K. Vinet J. Boddeke H. W. (2011). Microglia phenotype diversity. *CNS Neurol. Disord. Drug Targets* 10 108–118. 10.2174/187152711794488575 21143141

[B25] Papazian O. (2018). Neonatal hypoxic-ischemic encephalopathy. *Medicina* 78(Suppl. 2), 36–41. 30199363

[B26] Park J. H. Noh Y. Kim S. Ahn J. H. Ohk T. G. (2018). Time-course changes and new expressions of MIP-3 and its receptor, CCR6, in the gerbil hippocampal CA1 area following transient global cerebral ischemia. *Neurochem. Res.* 43 2102–2110. 10.1007/s11064-018-2632-6 30203401

[B27] Patel A. R. Ritzel R. McCullough L. D. Liu F. (2013). Microglia and ischemic stroke: a double-edged sword. *Int. J. Physiol. Pathophysiol. Pharmacol.* 5 73–90. 23750306 PMC3669736

[B28] Paudel Y. N. Shaikh M. F. Chakraborti A. Kumari Y. Aledo-Serrano A. (2018). HMGB1: a common biomarker and potential target for TBI, neuroinflammation, epilepsy, and cognitive dysfunction. *Front. Neurosci.* 12:628. 10.3389/fnins.2018.00628 30271319 PMC6142787

[B29] Qiu J. Nishimura M. Wang Y. Sims J. R. Qiu S. (2008). Early release of HMGB-1 from neurons after the onset of brain ischemia. *J. Cereb. Blood Flow Metab.* 28 927–938. 10.1038/sj.jcbfm.9600582 18000511

[B30] Sanches E. F. van de Looij Y. Toulotte A. Sizonenko S. V. Lei H. (2019). Mild neonatal brain Hypoxia-Ischemia in very immature rats causes Long-Term behavioral and cerebellar abnormalities at adulthood. *Front. Physiol.* 10:634. 10.3389/fphys.2019.00634 31231232 PMC6560160

[B31] Serdar M. Kempe K. Rizazad M. Herz J. Bendix I. (2019). Early pro-inflammatory microglia activation after inflammation-sensitized hypoxic-ischemic brain injury in neonatal rats. *Front. Cell Neurosci.* 13:237. 10.3389/fncel.2019.00237 31178702 PMC6543767

[B32] Sun Q. Wu W. Hu Y. C. Li H. Zhang D. (2014). Early release of high-mobility group box 1 (HMGB1) from neurons in experimental subarachnoid hemorrhage in vivo and in vitro. *J. Neuroinflammation* 11:106. 10.1186/1742-2094-11-106 24924349 PMC4107626

[B33] Sun X. Zeng H. Wang Q. Yu Q. Wu J. (2018). Glycyrrhizin ameliorates inflammatory pain by inhibiting microglial activation-mediated inflammatory response via blockage of the HMGB1-TLR4-NF-kB pathway. *Exp. Cell Res.* 369 112–119. 10.1016/j.yexcr.2018.05.012 29763588

[B34] Tang Z. Jiang M. Ou-Yang Z. Wu H. Dong S. Hei M. (2019). High mobility group box 1 protein (HMGB1) as biomarker in hypoxia-induced persistent pulmonary hypertension of the newborn: a clinical and in vivo pilot study. *Int. J. Med. Sci.* 16 1123–1131. 10.7150/ijms.34344 31523175 PMC6743282

[B35] Terrando N. Yang T. Wang X. Fang J. Cao M. (2016). Systemic HMGB1 neutralization prevents postoperative neurocognitive dysfunction in aged rats. *Front. Immunol.* 7:441. 10.3389/fimmu.2016.00441 27822212 PMC5075578

[B36] Thal S. E. Zhu C. Thal S. C. Blomgren K. Plesnila N. (2011). Role of apoptosis inducing factor (AIF) for hippocampal neuronal cell death following global cerebral ischemia in mice. *Neurosci. Lett.* 499 1–3. 10.1016/j.neulet.2011.05.016 21616126

[B37] Umekawa T. Osman A. M. Han W. Ikeda T. Blomgren K. (2015). Resident microglia, rather than blood-derived macrophages, contribute to the earlier and more pronounced inflammatory reaction in the immature compared with the adult hippocampus after hypoxia-ischemia. *Glia* 63 2220–2230. 10.1002/glia.22887 26179283 PMC5034822

[B38] Wang H. Bloom O. Zhang M. Vishnubhakat J. M. Ombrellino M. (1999). HMG-1 as a late mediator of endotoxin lethality in mice. *Science* 285 248–251. 10.1126/science.285.5425.248 10398600

[B39] Weinstein J. R. Koerner I. P. Moller T. (2010). Microglia in ischemic brain injury. *Future Neurol.* 5 227–246. 10.2217/fnl.10.1 20401171 PMC2853969

[B40] Wu Y. W. Mathur A. M. Chang T. McKinstry R. C. Mulkey S. B. (2016). High-Dose erythropoietin and hypothermia for Hypoxic-Ischemic encephalopathy: a phase II trial. *Pediatrics* 137:e20160191. 10.1542/peds.2016-0191 27244862

[B41] Xia C. Y. Zhang S. Gao Y. Wang Z. Z. Chen N. H. (2015). Selective modulation of microglia polarization to M2 phenotype for stroke treatment. *Int. Immunopharmacol.* 25 377–382. 10.1016/j.intimp.2015.02.019 25704852

[B42] Ye Y. Zeng Z. Jin T. Zhang H. Xiong X. Gu L. (2019). The role of high mobility group box 1 in ischemic stroke. *Front. Cell Neurosci.* 13:127. 10.3389/fncel.2019.00127 31001089 PMC6454008

[B43] Zhang J. Klufas D. Manalo K. Adjepong K. Davidson J. O. (2016). HMGB1 translocation after ischemia in the ovine fetal brain. *J. Neuropathol. Exp. Neurol.* 75 527–538. 10.1093/jnen/nlw030 27151753 PMC6366657

[B44] Zhang J. Takahashi H. K. Liu K. Wake H. Liu R. (2011). Anti-high mobility group box-1 monoclonal antibody protects the blood-brain barrier from ischemia-induced disruption in rats. *Stroke* 42 1420–1428. 10.1161/STROKEAHA.110.598334 21474801

[B45] Zhou X. Chu X. Xin D. Li T. Bai X. (2019). L-Cysteine-Derived H2S promotes microglia m2 polarization via activation of the AMPK pathway in Hypoxia-Ischemic neonatal mice. *Front. Mol. Neurosci.* 12:58. 10.3389/fnmol.2019.00058 30914921 PMC6421291

